# Recent FDA Approvals in the Treatment of Graft-Versus-Host Disease

**DOI:** 10.1093/oncolo/oyac076

**Published:** 2022-04-20

**Authors:** Dylan J Martini, Yi-Bin Chen, Zachariah DeFilipp

**Affiliations:** Department of Medicine, Massachusetts General Hospital, Boston, MA, USA; Hematopoietic Cell Transplant and Cellular Therapy Program, Massachusetts General Hospital, Boston, MA, USA; Hematopoietic Cell Transplant and Cellular Therapy Program, Massachusetts General Hospital, Boston, MA, USA

**Keywords:** graft-versus-host disease, ruxolitinib, ibrutinib, belumosudil, FDA approval

## Abstract

Graft-versus-host disease (GVHD) is a common complication of allogeneic hematopoietic cell transplantation (HCT) and is associated with significant morbidity and mortality. For many years, there have been few effective treatment options for patients with GVHD. First-line systemic treatment remains corticosteroids, but up to 50% of patients will develop steroid-refractory GVHD and the prognosis for these patients is poor. Elucidation of the pathophysiological mechanisms of acute and chronic GVHD has laid a foundation for novel therapeutic approaches. Since 2017, there have now been 4 approvals by the US Food and Drug Administration (FDA) for GVHD. Ruxolitinib, an oral selective JAK1/2 inhibitor, received FDA approval for the treatment of steroid-refractory acute GVHD in 2019 and remains the only agent approved for acute GVHD. There are currently 3 FDA approvals for the treatment of chronic GVHD: (1) ibrutinib, a BTK inhibitor traditionally used for B-cell malignancies, was the first agent approved for chronic GVHD after failure of one or more lines of systemic therapy, (2) belumosudil, an oral selective inhibitor of ROCK2, for patients with chronic GVHD who received at least 2 prior lines of treatment, and (3) ruxolitinib for chronic GVHD after failure of one or two lines of systemic therapy. In this review, we highlight the clinical data which support these FDA approvals in acute and chronic GVHD with a focus on mechanism of actions, clinical efficacy, and toxicities associated with these agents.

## Teaser

Considering the increasing number of allogeneic hematopoietic stem cell transplantations now being performed each year, non-transplant oncologists are more likely to encounter patients receiving treatment for graft versus host disease (GVHD) in their clinical practice. This review highlights recent approvals of ruxolitinib, ibrutinib, and belumosudil as therapeutic agents for GVHD, with a focus on mechanism of action, efficacy, and toxicity of these agents.

**Implications for Practice**

Graft-versus-host disease (GVHD) is a common and morbid complication of allogeneic hematopoietic cell transplantation (HCT). After many years without effective therapies, there have been 4 US Food and Drug Administration (FDA) approvals for GVHD treatment since 2017. In this review, we detail the efficacy and toxicity data from the clinical trials which support these approvals. As these newly approved agents are likely to be increasingly used in the clinical setting, this review has significant relevance for practicing oncologists treating patients after allogeneic HCT.

## Introduction

Allogeneic hematopoietic stem cell transplantation (HCT) is a potentially curative treatment for numerous malignant and nonmalignant hematologic and immunological diseases. However, HCT has traditionally been reserved for high-risk disease due to the risks associated with treatment. Graft-versus-host disease (GVHD), which affects 30%-50% of patients, is a major cause of morbidity and mortality in allogeneic HCT recipients.^1-3^ Additionally, many patients with GVHD suffer impairments in psychosocial function that result in significantly reduced quality of life (QoL).^4,5^ GVHD occurs when alloreactive donor cells (the graft) recognize the transplant recipient’s body (the host) as foreign, resulting in immunologically mediated damage to host tissues.^6^

Traditionally, therapeutic agents for GVHD have consisted of additional broad systemic immunosuppression, which have been associated with less than satisfactory response rates and resulted in an increased risk for opportunistic infection.^7^ Following preclinical investigations which further elucidated the pathophysiological mechanisms of both acute and chronic GVHD, the therapeutic landscape has shifted toward to the use of targeted agents with the hope of achieving improved clinical responses with less off-target effects.^8-10^ Since 2017, there have been 4 approvals by the US Food and Drug Administration (FDA) for GVHD: ruxolitinib (acute and chronic GVHD), ibrutinib (chronic GVHD), and belumosudil (chronic GVHD) (Table 1). Given the increasing number of allogeneic HCT being performed each year, nontransplant oncologists are more likely to encounter patients receiving treatment for GVHD in their clinical practice. In this review, we highlight these recent approvals with a focus on mechanism of action, efficacy, and toxicity associated with these agents.

**Table 1.** Overview of recent FDA-approved agents for GVHD

Agent	Mechanism of action	FDA-approved indication	Recommended starting dosage	Key toxicity considerations
Ruxolitinib	Selective inhibition of Janus kinases 1 and 2	Adult or pediatric patients 12 years and older with steroid-refractory acute GVHD	5 mg orally twice daily	Thrombocytopenia Anemia Neutropenia CMV infection Peripheral edema Sepsis Pulmonary Hemorrhage
		Adult or pediatric patients 12 years and older with chronic GVHD after failure of one or two lines of systemic therapy	10 mg orally twice daily	
Ibrutinib	Selective inhibition of Bruton’s tyrosine kinase	Adult patients with chronic GVHD after failure of one or more lines of systemic therapy	420 mg orally once daily	Fatigue Diarrhea Muscle spasms Pneumonia Atrial arrhythmias Fungal infections
Belumosudil	Selective inhibition of rho-associated coiled-coil-containing protein kinase-2	Adult or pediatric patients 12 years and older with chronic GVHD after failure of at least 2 prior lines of systemic therapy	200 mg orally once daily	Fatigue Diarrhea Nausea URI Pneumonia Hypertension Hyperglycemia Elevated Liver Function Tests

Abbreviations: CMV, cytomegalovirus; FDA, US Food and Drug Administration; GVHD, graft-versus-host disease; mg, milligram; URI, upper respiratory tract infection.

### GVHD Overview

Acute and chronic GVHD have unique pathophysiologies, clinical presentations, and risk factors, which are highlighted in Table 2.

**Table 2.** Pathophysiology, risk factors, and clinical presentation of acute and chronic GVHD.

GVHD type	Pathophysiology pathways^8,9,11^	Risk factors^12^	Clinical presentation
Acute GVHD	• Tissue damage from conditioning or infection • Recognition of foreign major and minor HLA antigens • Altered mechanisms of tissue repair and protection	• Degree of HLA mismatch • Female donors to male recipient • Total body irradiation	•** Skin:** maculopapular rash •** Gastrointestinal Tract:** nausea, vomiting, diarrhea •** Liver:** hyperbilirubinemia and jaundice
Chronic GVHD	• Acute inflammation and tissue injury • Chronic inflammation and dysregulated T-cell and B-cell immunity • Aberrant tissue repair and fibrosis	• Degree of HLA mismatch • Older patient age • Older donor age • Female donors to male recipient • Mobilized peripheral blood cell graft • Prior history of acute GVHD	*Can present with inflammatoryand/or fibrotic manifestations in the following organs:* •** Skin** •** Mouth** •** Eyes** •** Gastrointestinal Tract** •** Liver** •** Lungs** •** Joint/Fascia** •** Genital Tract**

Abbreviations: GVHD, graft versus host disease; HLA, human leukocyte antigen.

Generally, the pathophysiology of acute GVHD is thought to be initial injury to the recipient tissue, either from the conditioning regimen or infection, which ultimately leads to activation of alloreactive donor T cells which mediate tissue damage via direct attack or through propagation of inflammation. Acute GVHD is driven by recognition of mismatched major and minor histocompatibility antigens, altered mechanisms of tissue repair and protection, and loss of protective microbial-derived metabolites.^9,13^ While some aspects of the acute GVHD immune response overlap with chronic GVHD, the pathophysiology of chronic GVHD is distinct and can be summarized as having 3 phases: (1) host tissue injury which leads to early inflammation, (2) chronic inflammation and dysregulation of T-cell and B-cell immunity, and (3) tissue repair with fibrosis.^11^ Chronic GVHD biology is characterized by B-cell signaling and prolonged immune activation of T-cell subsets, regulatory T-cell deficiencies, and tissue fibrosis.^8^ Differences in the pathophysiological mechanism of acute and chronic GVHD form the basis for the development of unique prophylactic and therapeutic approaches to the disease.

While acute GVHD classically presents earlier after HCT than chronic GVHD, the distinction is defined by the symptoms at clinical presentation, rather than time of onset.^14^ The cardinal organ manifestations of acute GVHD are the skin (maculopapular rash), liver (hyperbilirubinemia and jaundice), and the gut (nausea, vomiting, diarrhea). Although chronic GVHD may involve any organ system, the most common organs involved are the skin, eyes, and mouth. One feature unique to chronic GVHD is the development of fibrotic changes, most notably sclerodermatous GVHD and bronchiolitis obliterans syndrome.^15^

Proven clinical risk factors for acute GVHD include degree of recipient human leukocyte antigen (HLA) mismatching, use of total body irradiation, and increased intensity of the conditioning regimen while risk factors more associated with chronic GVHD include older age, female donors to male recipients, prior history of acute GVHD, and peripheral blood stem cells as a graft source.^12^ Grading of disease severity remains clinically based with clear consensus criteria published for use in practice as well as in clinical trials for eligibility and response assessment.^16,17^ While noninvasive biomarkers are commercially available for acute GVHD risk stratification,^18,19^ no clinical trials have proven that these biomarkers should dictate choice of therapy. Chronic GVHD biomarkers are being actively investigated; however, none have yet emerged as being able to accurately risk stratify patients.^20^

## FDA Approved Agents for Acute GVHD

The standard approach to initial systemic treatment for acute GVHD is corticosteroids usually at a dose of 1-2 mg/kg/day of prednisone or its equivalent.^21-23^ However, approximately 50% of patients become steroid-resistant or refractory (SR),^24^ and these patients have a dismal long-term prognosis, with an estimated 40% nonrelapse mortality (NRM) rate within 12 months.^18^ Till date, no consensus has been reached regarding the optimal approach for the management of SR acute GVHD; however, the FDA approval of ruxolitinib for SR acute GVHD in May 2019 provides an avenue to a widely adopted approach to the initial management of SR acute GVHD.^25^

### Ruxolitinib

Ruxolitinib is a selective JAK 1/2 inhibitor that targets a class of intracellular kinases that have an important role in the development and function of immune cells including components of both the innate and adaptive immune system.^26^ The JAK signaling pathway is an important contributor of cytokine-driven tissue damage in acute GVHD, and preclinical models showed a reduction in incidence and severity of GVHD with the administration of JAK 1/2 inhibitors.^9,27-30^ The mechanism of immunomodulation induced by ruxolitinib is hypothesized to be via decreased neutrophil migration in the first phase of acute GVHD, decreased T-cell priming via downregulation of MHC-II and reduced cytokine release in the second phase, and reduced T-cell expansion in the third phase.^31^ The SR acute GVHD approval for ruxolitinib was based the results of REACH1, an open-label single-arm phase II trial which accrued subjects from December 2016 until July 2018 (Table 3).^32^

**Table 3.** Key clinical trials that led to the FDA approvals for acute and chronic GVHD

GVHD	Agent	Study	Number of subjects	Main eligibility Criteria	ORR at 28 days(CR)	Best ORR (CR)
Acute GVHD	Ruxolitinib	REACH1; Single arm Phase II	71	Age ≥ 12, any donor source for HCT, Grade II-IV steroid-refractory GVHD*, no more than 1 prior systemic treatment in addition to corticosteroids, myeloid engraftment	55% (27%)	73.2
		REACH 2; Randomized Phase III	309		62% (34%)	Not reported
Chronic GVHD	Ibrutinib	PYC-1129; Open-label, Phase Ib/II	42	Age ≥ 18, steroid-dependent or steroid-refractory GVHD, received no more than 2 previous regimens for GVHD, Erythematous rash >25% BSA or total National Institutes of Health (NIH) mouth score > 4	Not reported	67% (21%)
	Belumosudil	ROCKSTAR; Randomized Phase II	132	Age ≥ 12, persistent GVHD manifestations after 2-5 lines of systemic therapy, stable dose of corticosteroids for 2 weeks prior to enrollment	Not reported	76% (5%)
	Ruxolitinib	REACH 3; Randomized Phase III	329	Age ≥ 12, moderate-to-severe steroid-refractory or steroid dependent GVHD**, no more than 1 prior systemic treatment in addition to corticosteroids	50%*** (7%)	76% (12%)

Abbreviations: FDA, US Food and Drug Administation; GVHD, graft-versus-host disease; ORR, objective reponse rate; CR, complete response; HCT, hematopoietic cell transplantation; BSA, body surface area.

*Per Mount Sinai Acute GVHD International Consortium (MAGIC) criteria.

**According to NIH consensus criteria.

#### REACH1

REACH1 was a multicenter phase II trial which accrued subjects at 26 medical centers in the US. Eligible subjects included those who were at least 12 years of age with grades II-IV SR-GVHD per Mount Sinai Acute GVHD International Consortium (MAGIC) criteria,^16^ and receipt of <2 prior lines of systemic therapy for GVHD other than corticosteroids. SR criteria included participants who had progressive GVHD after 3 days of primary treatment or lack of improvement after 7 days of treatment with equivalent of at least 2 mg/kg methylprednisone, inability to taper corticosteroids, or development of newly involved organ system after initiation of low-dose corticosteroid treatment. The primary endpoint was 28-day overall response rate (ORR), which was defined as a complete response (CR), very good partial response (VGPR), or partial response (PR).

Approximately 71 subjects received at least 1 dose of ruxolitinib. The median age was 58 years and most participants (*n* = 48, 67.6%) had grade III-IV acute GVHD at enrollment. The median dose of corticosteroids at enrollment was 156.3 mg/day (range: 50-300) and the median duration of corticosteroid treatment prior to enrollment was 15 days (range: 3-285). The median average total daily dose of ruxolitinib was 10.3 mg/day (range: 5-20) and the median duration of treatment was 46 days (range: 4-473). The most common reasons for treatment discontinuation were adverse events (AEs, 28.2%), investigator discretion (28.2%), and death (9.9%). Only 8.5% of participants discontinued treatment for acute GVHD progression. More than half of subjects (54.9%) had a response at day 28, including 26.8% who experienced a CR. The median duration of response (DOR) was 345 days after ruxolitinib initiation. The ORR at any time for the entire cohort was 73.2%, including 4 subjects who responded after day 28 (1 CR, 3 PR). The only significant association with response in subgroup analysis was GVHD grade at enrollment, as participants with grade II GVHD had higher ORR compared to grade III and IV (82.6% vs. 41.2% vs. 42.9%). Indeed, there was a correlation in CR rate from participants with baseline grade II GVHD (47.8%) to grade III (20.6%) and grade IV (7.1%). Responses were observed across type of organs involved in GVHD; however, subjects with 2+ organs involved were less likely to have a response at 28 days compared to participants with 1 organ involved at enrollment (47.2% vs. 62.9%). The median overall survival (OS) was 7.6 months, with 6- and 12-month OS rates of 51% and 42.6%, respectively. The 6-month cumulative incidence rate for nonrelapse mortality (NRM) was 44.4% (95% CI, 32.5%-55.7%) and the 12-month cumulative rate for NRM was 52.9% (95% CI, 39.6%-64.5%) and both were lower for day 28 responders. Only 4 subjects were reported to have developed chronic GVHD after treatment with ruxolitinib.

Every participant enrolled onto the study experienced at least 1 treatment-emergent AE and 74.6% experienced at least 1 treatment-related AE with the most common being thrombocytopenia (Any grade: 47.9%, Grade 3 or 4: 42.3%), anemia (Any grade: 35.2%, Grade 3 or 4: 28.2%), and decreased neutrophil count (Any grade: 26.8%, Grade 3 or 4: 21.1%). Other AEs experienced by at least 10% of subjects included decreased white blood count (Any grade: 19.7%, Grade 3 or 4: 11.3%) and alanine aminotransferase (ALT) elevation (Any grade: 11.3%, Grade 3 or 4: 1.4). Toxicity led to ruxolitinib discontinuation, dose reduction, and treatment interruption in 32.4%, 35.2%, and 40.8% of participants, respectively. Grade 5 AEs were experienced by 2 patients (pulmonary hemorrhage and sepsis, both *n* = 1).

#### REACH2

The compelling results of the single-arm REACH1 trial were further confirmed by REACH2, a randomized international multicenter phase III trial comparing ruxolitinib versus investigators choice for therapy of SR acute GVHD (Table 3).^33^ Key eligibility criteria mirrored those for REACH1 as described above including subjects with grade II-IV SR acute GVHD who had received at most one prior systemic therapy other than steroids for acute GVHD. The primary endpoint was day 28 ORR and the key secondary endpoint was DOR at day 56.

A total of 309 participants were randomized from April 2017 until May 2019, with 154 subjects receiving ruxolitinib. The median age of enrolled participants was 54.0 years. The most common initial control treatment was extracorporeal photopheresis (27%).

In primary endpoint analysis, subjects who received ruxolitinib had significantly higher day 28 ORR compared to the control group (62% vs. 39%; Odds Ratio (OR): 2.64, *P* < .001) and were more likely to experience a CR (34% vs. 19%). Participants with grade II GVHD were most likely to experience an objective response in both groups (ruxolitinib: 75%; control group: 51%). Subjects with grade IV GVHD receiving ruxolitinib were more than twice as likely to have a response compared to the control group (53% vs. 23%; OR: 3.76). The DOR at 56 days was also significantly higher in the ruxolitinib group compared to the control group (40% vs. 22%; OR: 2.38, *P* < .0001) and the best overall response at day 28 was 82% in the ruxolitinib group and 61% in the control group (OR: 3.07, 95% CI, 1.80-5.25). Median OS and failure-free survival (FFS) also favored ruxolitinib (OS: 11.1 vs. 6.5 months, HR: 0.83, 95% CI 0.60-1.15; FFS: 5.0 vs. 1.0 months, HR: 0.46; 95% CI = 0.35-0.60).

Nearly all patients who received ruxolitinib (95%) experienced at least 1 AE, with 78% experiencing grade ≥3 AEs. Hematologic laboratory abnormalities were noted with similar frequency as in REACH1 with thrombocytopenia (Any grade: 50%, Grade ≥3: 41%), anemia (Any grade: 30%, Grade ≥3: 22%), and neutropenia (Any grade: 16%, Grade ≥3: 13%) being the most common. Other AEs observed in ≥10% of participants included cytomegalovirus infection, peripheral edema, hypokalemia, hypertension, hypoalbuminemia, pyrexia, and hypomagnesemia. AEs led to dose modification in 38% of subjects treated with ruxolitinib and 11% of participants discontinued ruxolitinib for AEs.

#### Acute GVHD: Summary and Critical Review

The results from REACH1 and REACH2 demonstrated that ruxolitinib is an effective treatment for SR acute GVHD with a tolerable toxicity profile. More than half of the subjects enrolled on these studies experienced an objective response at day 28 after treatment initiation (54.9%-62%), including more than one-quarter of subjects who experienced a CR (26%-33%). While REACH1 led to the FDA approval of ruxolitinib, REACH2 demonstrated significant improvement in efficacy outcomes with the use of ruxolitinib as compared to other current treatment options. We believe these studies established ruxolitinib as the standard of care for SR acute GVHD. However, real-world experiences with ruxolitinib will be of high interest. In practice, ruxolitinib may be initiated early as a steroid-sparing agent, before SR criteria from the clinical trials are met. Additionally, patients with grade III-IV disease at the time of enrollment on REACH2 only showed a 53%-56% response rate with ruxolitinib, illustrating that severe disease remains a significant unmet need. Future studies are needed to identify which patients are most likely to benefit from ruxolitinib, duration of therapy, how to taper and discontinue therapy, how to define ruxolitinib refractory disease and optimal treatments for those patients who do not have a satisfactory response to ruxolitinib.^34,35^

## FDA Approved Agents for Chronic GVHD

Similar to acute GVHD, corticosteroids have traditionally been the recommended first-line treatment for chronic GVHD.^23,36^ Although there is no consensus treatment for SR chronic GVHD, commonly used agents include calcineurin inhibitors, extracorporeal photopheresis (ECP), mammalian target of rapamycin (mTOR) inhibitors, rituximab, and mycophenolate mofetil. There have been 3 recent approvals for the treatment of refractory chronic GVHD: ibrutinib, belumosudil, and ruxolitinib.

### Ibrutinib

Ibrutinib is an oral selective and irreversible inhibitor of Bruton’s tyrosine kinase (BTK), which inhibits signal transduction from the B-cell receptor, activation of B-cells, and interleukin-2-inducible T-cell kinases (ITK).^37^ Pre-clinical models found that mice who underwent HCT from BTK- or ITK-deficient donors did not develop chronic GVHD, suggesting a possible role for ibrutinib for the treatment of chronic GVHD.^38^ Ibrutinib became the first FDA-approved agent for chronic GVHD in 2017 based on the results of a single-arm Phase II trial (PYC-1129) (Table 3).

#### PCYC-1129

This clinical trial enrolled subjects across 11 centers in the US. Participants were eligible for the trial if they were ≥18 years old with steroid-dependent or SR chronic GVHD and had ≤ 3 prior systemic treatment regimens. Steroid-dependent disease was defined as GVHD requiring at least 12 weeks of prednisone ≥ 0.25 mg/kg, while SR disease was defined as progression of chronic GVHD after at least 4 weeks of treatment with ≥ 0.5 mg/kg prednisone. Patients were eligible if they had active chronic GVHD, defined as having an erythematous rash involving at least 25% of body surface area or a NIH mouth score >4. The requirement of one of these inflammatory manifestations of chronic GVHD has not been adopted in subsequent trials. The primary efficacy endpoint for the phase II portion of the trial was best ORR at any time, defined as a CR or PR based on the 2005 NIH Chronic GVHD Consensus Panel and modified based on the 2014 NIH response criteria.^39,40^

A total of 42 subjects received 420 mg ibrutinib daily. The median age was 56 years and most participants previously received nonmyeloablative transplants (57%). The median time from transplant to chronic GVHD diagnosis was 7.6 months and the median time from chronic GVHD diagnosis to treatment with ibrutinib was 13.7 months. Most subjects (57%) had 2 organs involved in GVHD and 28% had ≥3 organs involved. The median time on treatment with ibrutinib was 4.4 months.^41^ The ORR for the entire cohort was 67%, including 21% of participants who experienced a CR. Of the subjects who responded, 79% showed a response at their first assessment and 71% experienced a sustained response for ≥ 20 weeks. Organ-specific subgroup analysis demonstrated high response rates in the skin (88%) and mouth (99%). Participants with steroid-dependent GVHD had higher response rates compared to SR or both steroid-dependent and SR subjects (75% vs. 50% vs. 50%). One-year follow-up data showed 55% of patients had sustained responses for >44 weeks.^41^ The median corticosteroid dose decreased on ibrutinib from a baseline of 0.29-0.12 mg/kg at week 49 and 5 participants discontinued steroids during treatment with ibrutinib. Nearly one-quarter of subjects (24%) experienced at least a 7-point decrease in the Lee Symptoms Scale (LSS).

The majority of treatment-related AEs were grade 1-2 and the most common were fatigue (*n* = 24, 57%), diarrhea (*n* = 37, 37%), and muscle spasms (*n* = 12, 28%). The most common serious adverse events (grade ≥ 3) were fatigue (*n* = 5, 12%), pneumonia (*n* = 4, 10%), and diarrhea (*n* = 4, 10%). The only cardiac toxicity was atrial fibrillation, which was reported in one patient. Infectious complications were seen in 69% of participants and 2 subjects experienced fatal treatment-related AEs (pneumonia, bronchopulmonary aspergillosis). Toxicity led to dose reductions and treatment discontinuation in 31% and 33% of participants, respectively.

### Belumosudil

Belumosudil is an oral selective inhibitor of rho-associated coiled-coil-containing protein kinase-2 (ROCK2), which is an important signaling pathway that regulates Th17/regulatory T-cell balance and the profibrotic pathway.^42^ Belumosudil reduces Th17 and follicular helper cells via downregulation of STAT3 and enhances regulatory T cells via upregulation of STAT5. It also has the potential to downregulate profibrotic gene expression, which may inhibit the differentiation of fibroblasts to myeloblasts and decrease collagen production. Hence, this agent has a unique target in the treatment of chronic GVHD given that it targets both inflammation and fibrosis. In preclinical models, belumosudil was shown to significantly reduce lung and skin fibrosis in animal models which supported the hypothesis that ROCK2 inhibition may be an effective treatment in chronic GVHD, whose pathological hallmark is fibrosis.^43^ A phase II, open-label, dose finding study of belumosudil in patients with chronic GVHD who received 1-3 prior lines of systemic therapy showed promising results.^44^ The ORR at any time was 65%, including 60% in patients with severe chronic GVHD, and a median time-to-next-treatment of 14 months. Importantly, 50% of patients experienced a clinically meaningful improvement in their quality of life, defined as a decrease of ≥7 points in their LSS. FDA approval was subsequently granted for belumosudil in chronic GVHD in July 2021 based on results of a randomized phase II study (ROCKstar) of 2 different dosing schedules of belumosudil (Table 3).^45^

#### ROCKstar

This clinical trial enrolled subjects at 28 centers across the US. Important eligibility criteria included age ≥ 12 years, ongoing chronic GVHD manifestations, 2-5 prior lines of systemic therapy, and stable dose of corticosteroids for 2 weeks prior to screening.

In total, 132 subjects were enrolled onto the study and randomized 1:1 to receive either 200 mg QD or 200 mg BID of belumosudil. Subjects were treated until unacceptable toxicity or clinically significant progression of chronic GVHD on treatment. Best ORR at any time, defined as the rate of participants experiencing either CR or PR per 2014 NIH Consensus Criteria was the primary endpoint of the study.

The median age of enrolled subjects was 56 years and the majority of subjects had either moderate (31%) or severe (67%) GVHD at the time of enrollment. More than one-half (52%) of participants had ≥ 4 organs involved. In this heavily pre-treated population, 72% of subjects received ≥ 3 prior lines of systemic therapy including 34% who received ibrutinib and 29% who received ruxolitinib. The median duration of belumosudil treatment was 10 months, with 44% staying on treatment for ≥ 1 year. The most common reasons for treatment discontinuation were progression of disease (15.9%), AEs (12.1%), voluntary withdrawal (9.8%), and physician discretion (8.3%). The ORR was similar between the QD (74%, 95% CI: 62%-84%) and BID dosing groups (77%, 95% CI: 65%-87%). The CR rate of all subjects was 5.3% (*n* = 7). The ORR was similar for participants who received prior ibrutinib (74%, 95% CI: 59%-86%) or ruxolitinib (68%, 95% CI: 51%-83%). During treatment with belumosudil, 65% of subjects reduced their corticosteroid dose. Additionally, a clinically meaningful improvement in the LSS summary score from baseline was observed in 59% and 62% of subjects in the 200 mg daily and 200 mg twice daily cohorts.

Of all subjects enrolled into the study, 67% experienced a drug-related AE and 38% experienced a serious AE. The most common AEs were fatigue (38%), diarrhea (33%), nausea (31%), and cough (28%). Liver-related AEs were also reported in 24% of participants. Grade ≥3 AEs included pneumonia (8%), hypertension (6%), and hyperglycemia (5%).

### Ruxolitinib

Ruxolitinib was also recently granted FDA approval for SR chronic GVHD based on results from a phase III randomized control trial of ruxolitinib versus investigator’s choice for SR chronic GVHD (REACH 3) (Table 3).^46^

#### REACH3

Participants were enrolled onto this international trial at 49 centers across 28 countries in the US, Europe, Asia, Canada, and Australia. Eligible subjects were ≥ 12 years of age who had previously undergone allogenic HCT and subsequently developed moderate-to-severe SR or steroid-dependent chronic GVHD per NIH consensus criteria. Subjects were excluded if they received 2 or more systemic therapies for chronic GVHD in addition to steroids and they were eligible if they previously received a JAK inhibitor for acute GVHD if they met the following criteria: (1) had a PR or CR to prior JAK inhibitor treatments and (2) JAK inhibitor had been discontinued at least 8 weeks prior to enrollment onto the trial. The primary endpoint was objective response at 24 weeks, defined as a CR or PR per NIH criteria.

In total, 329 subjects were randomized. Most participants (61.1%) were male and the median age was 49 years. More than half (56.5%) had severe disease, while 42.9% had moderate disease. The most common investigator choice agents used as control therapy were ECP (34.8%), mycophenolate mofetil (22.2%), and ibrutinib (17.1%). The ORR at 24 weeks was significantly higher in the ruxolitinib group compared to best available therapy (49.7% vs. 25.6%, *P* < .001). More subjects in the ruxolitinib group had a CR compared to the control therapy group (6.7% vs. 3.0%). The best overall response was also significantly higher in the ruxolitinib group compared to the control group (76.4% vs. 60.4%, *P* = .001). Participants receiving ruxolitinib also had longer FFS (>18.6 vs. 5.7 months, *P* < .001) and higher Modified LSS response rate (24.2% vs. 11.0%, *P* < .001) compared to the control group. The ruxolitinib group was more likely to discontinue treatment for toxicity (17.0% vs. 4.9%) and less likely to discontinue for lack of efficacy (14.5% vs. 42.7%).

Similar to REACH1 and REACH2, common adverse events were anemia (29.1%), thrombocytopenia (21.2%), and neutropenia (10.9%). Other AEs experienced by >10% of subjects were pneumonia (10.9%), diarrhea (10.3%), ALT elevation (15.2%), elevated creatinine (13.9%), hypertension (15.8%), pyrexia (15.8%), cough (10.3%), and fatigue (10.3%).

### Summary and Critical Review

The FDA approvals of ibrutinib, belumosudil, and ruxolitinib over the last 5 years represent a monumental achievement for chronic GVHD therapeutics that will hopefully translate into improved clinical outcomes for patients. All 3 agents have shown clinically meaningful responses, characterized by high ORRs (67%-76%) and improvements in QoL. CRs have been generally rare in these studies, due to the nature of chronic GVHD itself. Ibrutinib had the highest reported CR rate (21%), although this may be in part due to more than half of the subjects in that trial having only 2 organs involved in their GVHD and cross-trial comparison is not appropriate due to differences in patient selection. Thus, we believe there is no consensus standard of care regarding choice of therapy for SR chronic GVHD, given the lack of head-to-head comparison (Table 4). Practically, the selection of therapeutic agent will be influenced by clinician familiarity, side effect profile, and accessibility. It will be important to evaluate the efficacy of these agents as they are implemented into clinical practice. Real world responses can be more modest than what is achieved in clinical trials.^47^ Furthermore, questions remain about how these agents will be used in clinical practice. Clinical trials till date have not allowed combined administration of these novel agents, which will inevitably occur. Reports on the safety and clinical efficacy of this approach will be of particular interest.

**Table 4.** Strengths and limitations of clinical investigation supporting recent FDA-approved agents for GVHD.

Strengths
• Strong pre-clinical data demonstrating biological rationale in GVHD
• Investigation of agents with different mechanisms of actions
• Multicenter prospective clinical trials, including 2 international studies
• Use of validated diagnostic criteria and response measures in clinical trials
Limitations
• Limited use of randomized, phase III design
• No head-to-head comparison of single agents
• Some trials with small to moderate sample size
• Scarce real-world experience reported to date

## Future Directions

Ongoing studies for GVHD are investigating novel agents, combination therapies, and steroid-sparing approaches. Given the numerous pathophysiologic mechanisms at play in both acute and chronic GVHD, there is a great opportunity to develop novel treatments, which remain an unmet need for this population. While many clinical trials are ongoing, a few select agents are being investigated in larger studies. Alpha-1-Antitrypsin (AAT), a serine protease inhibitor with anti-inflammatory and immunomodulatory properties, has previously demonstrated good tolerability and clinical efficacy as a treatment for SR acute GVHD.^48^ BMT CTN 1705 is an ongoing randomized phase III trial that will compare the use of AAT with corticosteroids to corticosteroids alone as first-line therapy for subjects with high risk acute GVHD (NCT04167514). T-guard, a combined CD3/CD7 immunotoxin which can inhibit both activated T-cell and NK cell function, will be compared to ruxolitinib in a randomized phase III trial in the treatment of SR Grade III-IV acute GVHD (BMT CTN 2002; NCT04934670).^49^ Similarly, a phase III trial of itolizumab, a monoclonal antibody targeting CD6-ALCAM pathway, in the up-front treatment of acute GVHD is planned, after demonstrating preliminary safety and efficacy in a phase Ib/II study.^50^ Axatilimab, a monoclonal antibody that targets CSF1-R to address aberrant macrophage function, is being investigated as therapy for SR chronic GVHD. Following a phase I/II study demonstrating tolerability and efficacy,^51^ an international, randomized phase II registration study for subjects with refractory chronic GVHD who have received at least 2 prior treatments has begun enrollment (NCT04710576). Aside from immune-targeted approaches to GVHD, the microbiome has emerged as a target for intervention, as lower intestinal microbial diversity during allogeneic HCT is associated with higher mortality and risk of death from acute GVHD.^52,53^ Fecal microbiome transplantation (FMT) can reverse dysbiosis after transplant,^54^ and preliminary studies have shown this approach to be clinically effective in treating SR acute GVHD.^55,56^ Although infection transmission is a possible risk with FMT,^57^ these events are likely rare.^58^ Larger multicenter studies are ongoing and planned to better evaluate the clinical efficacy of this microbiome-targeted approaches for SR acute GVHD (NCT03359980, NCT04769895).

While many clinical trials have targeted SR-GVHD, therapeutic agents are now being investigated in the upfront setting, acknowledging the suboptimal response to corticosteroids. Three recent large clinical trials have added investigational agents to corticosteroids in the front-line setting, in addition to BMT CTN 1705 mentioned above. In a randomized phase III trial, the addition of itacitinib, a selective JAK1 inhibitor, to corticosteroids did not significantly improve Day 28 ORR for grade II-IV acute GVHD as compared to corticosteroids with placebo (NCT03139604).^59^ Similarly, in chronic GVHD, the addition of ibrutinib in the front-line treatment of moderate/severe disease failed to improve response rates as compared to placebo when used in combination with corticosteroids (NCT02959944).^60^ An ongoing phase III trial is randomizing patients with newly diagnosed moderate or severe chronic GVHD to treatment with itacitinib or placebo in combination with corticosteroids (NCT03584516).

Furthermore, clinical trials in GVHD are striving to develop more individualized approaches to treatment. In acute GVHD, combinations of clinical risk scores and blood biomarkers are now being used to risk stratify patients with either higher- or lower-risk disease. In the setting of high-risk GVHD, the addition of other therapeutic agents to systemic corticosteroids for upfront treatment is being investigated (NCT02133924). In patients with lower-risk acute GVHD, trials are now investigating whether specific agents can be used that limit corticosteroid use or avoid them altogether. The theoretical benefits of such studies include limiting the toxicity of corticosteroids and creating an opportunity to better elucidate the biological effect of targeted monotherapy.^58^ In BMT CTN 1501, sirolimus demonstrated similar treatment efficacy and overall lower toxicity profile as compared to prednisone for standard risk GVHD, defined by the Minnesota GVHD Risk Score and MAGIC biomarker status.^61^ There are currently 2 clinical trials investigating this approach in chronic GVHD: ibrutinib as monotherapy (NCT04294641) and the combination of itacitinib and ECP (NCT04446182). Additionally, select GVHD therapeutics are targeted to specific clinical manifestations. For example, in acute gastrointestinal GVHD, interventions such as FMT^55,56^ and anti-α4-integrins, such as natalizumab and vedolizumab,^62,63^ have demonstrated organ-specific clinical efficacy. Similar approaches are of high interest for patients with fibrotic manifestations of chronic GVHD.^64^

## Conclusions

With an increasing number of allogeneic HCT being performed each year, nontransplant oncologists are more likely to be involved in the management of patients with ongoing GVHD. While the recommended frontline systemic therapy for both acute and chronic GVHD remains corticosteroids, there has long been no consensus on the treatment of SR-GVHD. There are now 3 agents approved for SR-GVHD: ruxolitinib (acute and chronic), ibrutinib (chronic), and belumosudil (chronic). The results of REACH2 make a compelling case that ruxolitinib should be considered the consensus first choice in the treatment for acute SR-GVHD and its uptake into clinical practice is increasing. In chronic GVHD, all 3 agents have shown good efficacy and favorable toxicity profiles in clinical trials. Future studies will continue to investigate these agents and other novel therapies in multiple settings and combinations and are sure to further advance the therapeutic landscape for GVHD.

## Data Availability

The data underlying this article will be shared at reasonable request to the corresponding author.
